# Supplementary material for: Astrocyte-Derived Small Extracellular Vesicles Regulate Dendritic Complexity through miR-26a-5p Activity
Source: Cells. 2020 Apr 10;9(4):930. doi: 10.3390/cells9040930 (PMC7226994; doi:10.3390/cells9040930)
Supplement: Supplementary file 1 [file cells-09-00930-s001.zip › Supplementary files/Supplementary figure 3.docx]

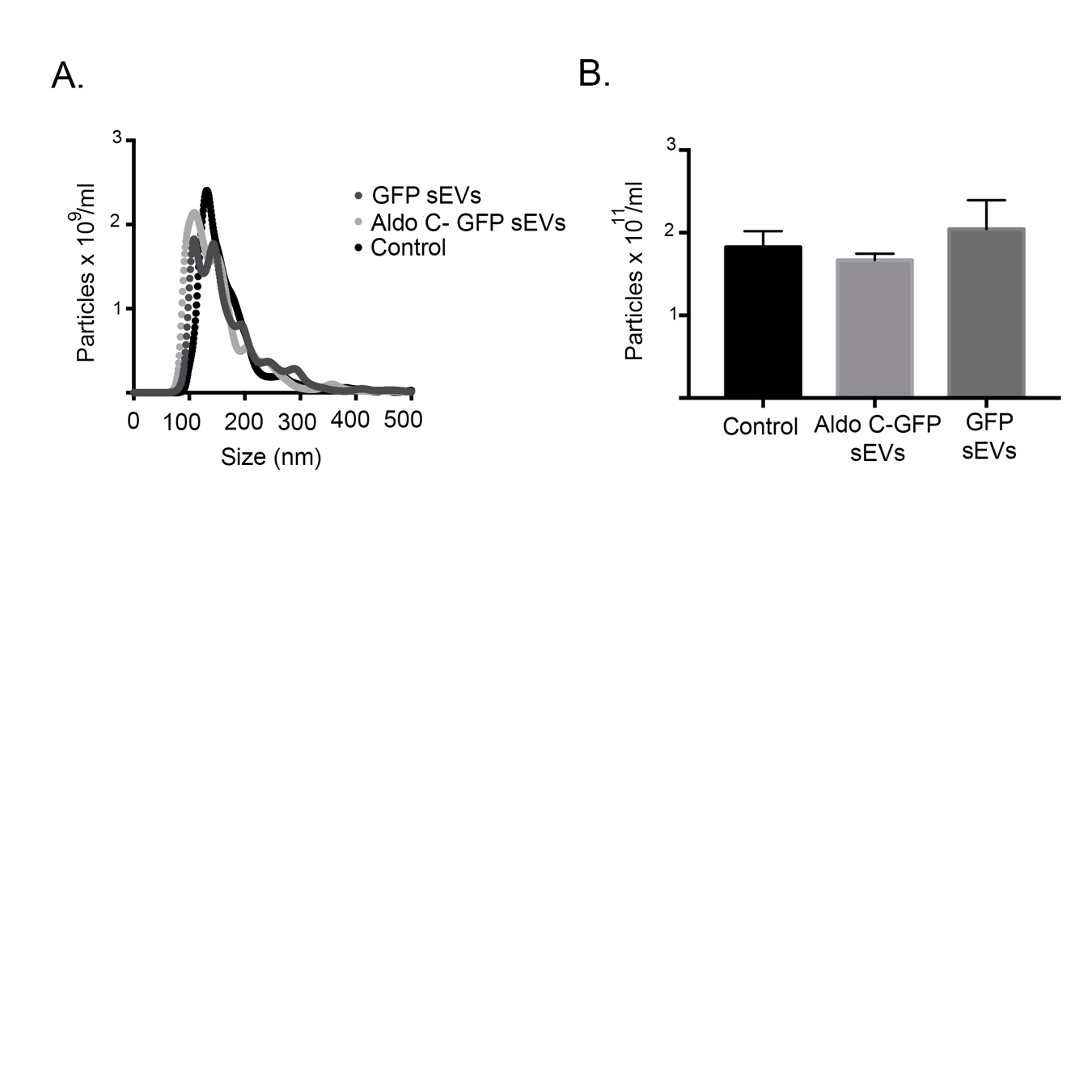


**Supplementary figure 3. Quantification of sEVs by NTA. A.** Size profile, i.e. histogram of the number of particles per particle diameter obtained by NTA, obtained from the indicated experimental conditions. The mean of N= 3-4 independent experiments is shown. **B.** The total concentration of particles is similar in the three experimental conditions.. Mean ± standard error of N= 3-4 independent experiments. No significant changes among the groups were found using one-way ANOVA followed by Tukey’s multiple comparison test.
