# Supplementary material for: Astrocyte-Derived Small Extracellular Vesicles Regulate Dendritic Complexity through miR-26a-5p Activity
Source: Cells. 2020 Apr 10;9(4):930. doi: 10.3390/cells9040930 (PMC7226994; doi:10.3390/cells9040930)
Supplement: Supplementary file 1 [file cells-09-00930-s001.zip › Supplementary files/Supplementary figure 2 .docx]

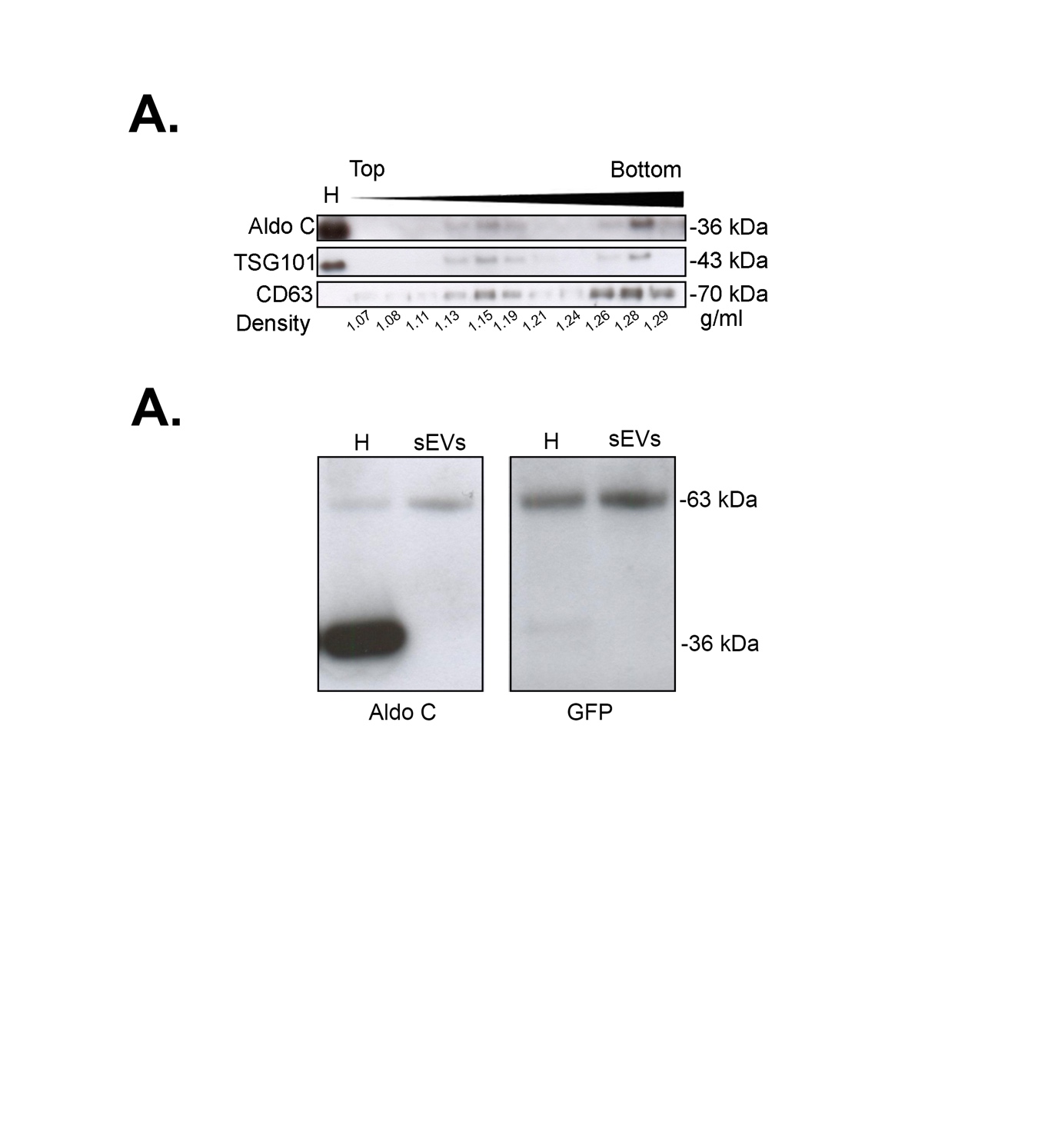


**Supplementary figure 2. Aldolase C co-distributes with sEVs markers of endosomal origin in fractions isolated from a sucrose gradient.** Representative Western blots from astrocyte-isolated fraction of sEVs loaded at the bottom of a sucrose gradient and centrifugated until flotation of different to corresponding densities. Densities (g/ml) of collected fractions and astrocyte homogenate (H) are indicated. Blots showed presence of two peaks of typical sEVs markers, including CD63, TSG101 in coincidence with Aldo C bands at densities compatible with sEVs. Images are representative of 3 independent experiments.
