# Supplementary material for: Astrocyte-Derived Small Extracellular Vesicles Regulate Dendritic Complexity through miR-26a-5p Activity
Source: Cells. 2020 Apr 10;9(4):930. doi: 10.3390/cells9040930 (PMC7226994; doi:10.3390/cells9040930)
Supplement: Supplementary file 1 [file cells-09-00930-s001.zip › Supplementary files/Supplementary table 1.docx]

|  |  |  | Aldo C-GFP/GFP |
| --- | --- | --- | --- |
| H samples | **Ct U6** | **Ct miR-26a-5p** | **Fold change (2^-ddCT)** |
| Aldo C-GFP 1 | 21,18 | 19,61 | 6,7 |
| GFP 1 | 19,72 | 20,89 |  |
| Aldo C-GFP 2 | 20,91 | 20,27 | 3,9 |
| GFP 2 | 19,91 | 21,22 |  |
| Aldo C-GFP 3 | 20,07 | 20,61 | 2,7 |
| GFP 3 | 19,66 | 21,61 |  |
| Aldo C-GFP 4 | 19,63 | 20,41 | 1,5 |
| GFP 4 | 19,98 | 21,35 |  |
| Aldo C-GFP 5 | 19,00 | 18,50 | 1,1 |
| GFP 5 | 19,25 | 18,90 |  |
| Aldo C-GFP 6 | 16,27 | 17,98 | 0,8 |
| GFP 6 | 16,54 | 17,99 |  |

A.

B.

|  |  |  | Aldo C-GFP/GFP |
| --- | --- | --- | --- |
| sEV samples | **Ct U6** | **Ct miR-26a-5p** | **Fold change (2^-ddCT)** |
| Aldo C-GFP 1 | 37,01 | 32,34 | 134,4 |
| GFP 1 | 26,12 | 28,52 |  |
| Aldo C-GFP 2 | 25,41 | 24,22 | 71,5 |
| GFP 2 | 24,93 | 29,90 |  |
| Aldo C-GFP 3 | 34,13 | 28,02 | 3,7 |
| GFP 3 | 31,22 | 27,01 |  |
| Aldo C-GFP 4 | 34,50 | 33,10 | 3,6 |
| GFP 4 | 33,60 | 33,90 |  |
| Aldo C-GFP 5 | 29,14 | 20,46 | 2,4 |
| GFP 5 | 32,33 | 24,94 |  |
| Aldo C-GFP 6 | 23,97 | 23,01 | 0,4 |
| GFP 6 | 29,28 | 26,99 |  |
| Aldo C-GFP 7 | 33,27 | 32,00 | 0,2 |
| GFP 7 | 32,47 | 28,70 |  |

**Supplementary Table 1. Individual experimental values of figure 3C.** **A.** RT-qPCR Cycle threshold (Ct) of miR26a-5p and U6 with the corresponding fold change value of miR26a-5p in cell homogenates from Aldo C-GFP astrocytes compared to GFP astrocytes at indicated samples (H)**.** **B.** Ct of miR26a-5p and U6 with the corresponding fold change value of miR26a-5p in Aldo C-GFP sEVs compared to GFP sEVs at indicated samples (sEV ). To obtain the fold change value all data set were corrected by U6 content using the double delta Ct method (2^-ddCT).
