# Supplementary material for: Astrocyte-Derived Small Extracellular Vesicles Regulate Dendritic Complexity through miR-26a-5p Activity
Source: Cells. 2020 Apr 10;9(4):930. doi: 10.3390/cells9040930 (PMC7226994; doi:10.3390/cells9040930)
Supplement: Supplementary file 1 [file cells-09-00930-s001.zip › Supplementary files/Supplementary figure 1 .docx]

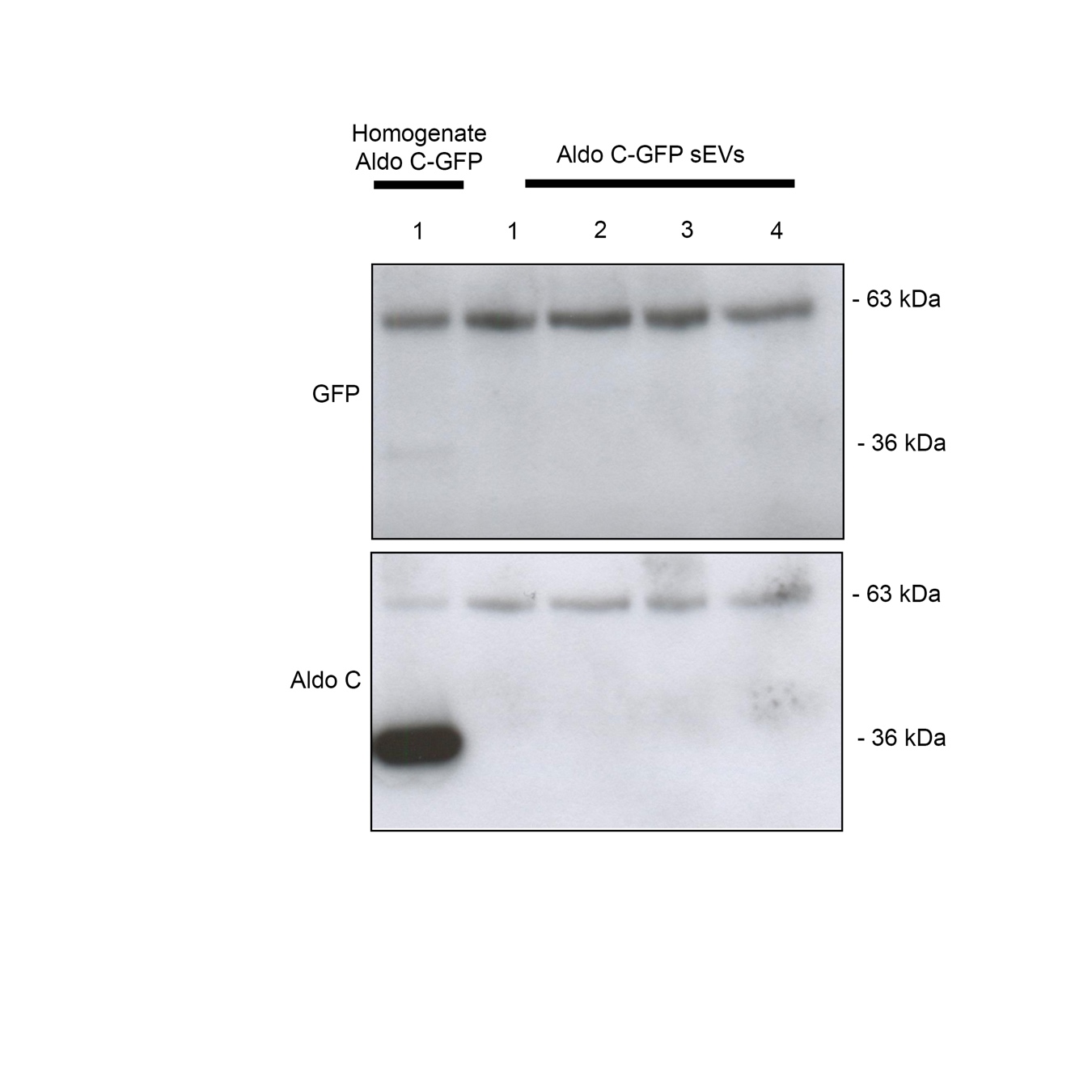


**Supplementary figure 1.** **Complete Western blot of figure 1E from the original manuscript.** The membrane was blotted using an GFP antibody. Homogenates of astrocytes expressing Aldo C-GFP showed one single band corresponding to the recombinant protein at 63KDa. When using an Aldo C antibody, two bands corresponding to the recombinant and endogenous proteins appeared. Lanes 1-4 are Aldo C-GFP sEVs from four independent astrocyte cultures. Equal amount of protein was loaded in all the lanes.
